# 37. Allogeneic, Off-the-Shelf, SARS-CoV-2-specific T Cells Demonstrate Reactivity Against Emerging Variant Strains

**DOI:** 10.1093/ofid/ofab466.037

**Published:** 2021-12-04

**Authors:** Spyridoula Vasileiou, Manik Kuvalekar, Aster Workineh, Ayumi Watanabe, Yovana Velazquez, Suhasini Lulla, Helen Elisabeth Heslop, Kimberly Mooney, Kevin Grimes, George Carrum, LaQuisa Hill, Premal Lulla, Ann Marie Leen

**Affiliations:** 1 Center for Cell and Gene Therapy, Baylor College of Medicine, Texas Children’s Hospital, Houston Methodist Hospital, Houston, TX; 2 AlloVir Inc, Houston, Texas

## Abstract

**Background:**

The impact of COVID-19 has been profound with >170,000,000 confirmed cases worldwide and emerging variants being a cause of global concern. Defects in T-cell function and trafficking have been described among those with severe illness, and immunodeficiency is a risk factor for persistent viral shedding and prolonged symptoms. Because of our prior clinical data demonstrating that allogeneic, off-the-shelf virus-specific T cells (VSTs) can safely and effectively treat viral infections, we investigated the feasibility of targeting COVID-19 using banked, SARS-CoV-2-specific VSTs.

**Methods:**

We first screened PBMCs from convalescent individuals against 18 structural and non-structural/accessory (NSPs/APs) SARS-CoV-2 proteins and identified 5 [Spike (S), Membrane (M), Nucleoprotein (N), NSP4, and AP7a] as immunodominant which were then advanced to our VST production process.

**Results:**

Using overlapping peptide libraries spanning these antigens as a stimulus, we achieved a mean 7.6±0.9 fold expansion (n=13) of VSTs (96±0.5%), with a mixture of cytotoxic (CD8+) and helper (CD4+) T cells that expressed activation and central/effector memory markers. These VSTs were potent, Th1-polarized and polyfunctional, producing IFNγ, TNFα, GM-CSF and Granzyme B. Moreover, the VSTs were able to kill pepmix-loaded autologous targets with no evidence of auto- or alloreactivity, attesting to their virus selectivity and safety for clinical use (Figure 1). Finally, though initially generated against the reference strain NC_045512.2 (Wuhan), these VSTs were able to recognize other clinically important variants including B1.1.7 (UK), B1.351 (South Africa) and P1 (Brazil). This demonstrates the cross-reactive potential of these polyclonal and diverse VSTs, which were developed to provide potent antiviral effects and minimize the risk of immune escape due to sequence variation.

Figure 1: SARS-CoV-2 Specific T cells Have Demonstrated Selective Cytolytic Activity against SARS-CoV-2 While Leaving Non-Virus Infected Targets Intact.

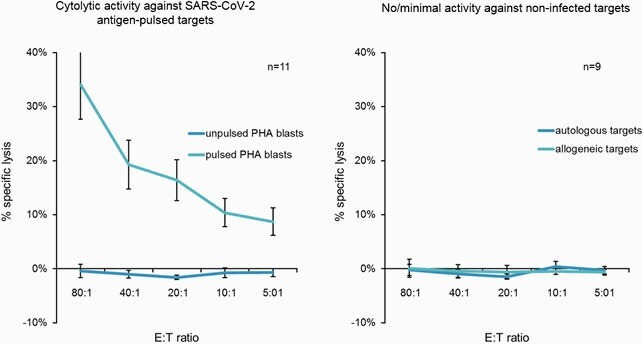

**Conclusion:**

In conclusion, it is feasible to generate polyclonal SARS-CoV-2 VSTs that provide coverage against variant strains using GMP-compliant manufacturing methodologies. We have advanced this product to the bedside for administration in a Phase I, randomized clinical trial [VSTs+ standard of care (SOC) vs SOC] in high-risk patients hospitalized with COVID-19 (NCT04401410).

**Disclosures:**

**Spyridoula Vasileiou, PhD**, **AlloVir** (Consultant) **Manik Kuvalekar, MSc**, **AlloVir** (Consultant) **Aster Workineh, MSc**, **AlloVir** (Employee) **Ayumi Watanabe, BSc**, **AlloVir** (Consultant) **Yovana Velazquez, BSc**, **AlloVir** (Consultant) **Helen Elisabeth Heslop, MD**, **AlloVir** (Shareholder)**Cell Medica** (Grant/Research Support)**Gilead Sciences** (Consultant)**Kiadis Pharma** (Consultant)**Marker Therapeutics** (Consultant, Shareholder)**Mesoblast** (Consultant)**Novartis** (Consultant)**PACT Pharma** (Consultant)**Tessa Therapeutics** (Consultant, Research Grant or Support) **Premal Lulla, MD**, **Johnson & Johnson** (Shareholder) **Ann Marie Leen, PhD**, **AlloVIr** (Consultant, Shareholder)**Marker Therapeutics** (Consultant, Shareholder)

